# Outlier Removal and the Relation with Reporting Errors and Quality of Psychological Research

**DOI:** 10.1371/journal.pone.0103360

**Published:** 2014-07-29

**Authors:** Marjan Bakker, Jelte M. Wicherts

**Affiliations:** 1 Department of Psychology, University of Amsterdam, Amsterdam, The Netherlands; 2 Tilburg School of Social and Behavioral Sciences, Tilburg University, Tilburg, The Netherlands; Copenhagen University Hospital Gentofte, Denmark

## Abstract

**Background:**

The removal of outliers to acquire a significant result is a questionable research practice that appears to be commonly used in psychology. In this study, we investigated whether the removal of outliers in psychology papers is related to weaker evidence (against the null hypothesis of no effect), a higher prevalence of reporting errors, and smaller sample sizes in these papers compared to papers in the same journals that did not report the exclusion of outliers from the analyses.

**Methods and Findings:**

We retrieved a total of 2667 statistical results of null hypothesis significance tests from 153 articles in main psychology journals, and compared results from articles in which outliers were removed (N = 92) with results from articles that reported no exclusion of outliers (N = 61). We preregistered our hypotheses and methods and analyzed the data at the level of articles. Results show no significant difference between the two types of articles in median *p* value, sample sizes, or prevalence of all reporting errors, large reporting errors, and reporting errors that concerned the statistical significance. However, we did find a discrepancy between the reported degrees of freedom of *t* tests and the reported sample size in 41% of articles that did not report removal of any data values. This suggests common failure to report data exclusions (or missingness) in psychological articles.

**Conclusions:**

We failed to find that the removal of outliers from the analysis in psychological articles was related to weaker evidence (against the null hypothesis of no effect), sample size, or the prevalence of errors. However, our control sample might be contaminated due to nondisclosure of excluded values in articles that did not report exclusion of outliers. Results therefore highlight the importance of more transparent reporting of statistical analyses.

## Introduction

The sharing of data for verification purposes is not common practice in psychology [1], [2] and other research fields [3]–[11]. Wicherts et al. [1] found that 73% of the contacted authors who had published a paper in one of four top journals published by the *American Psychological Association* (APA) failed to share their data, notwithstanding the fact that authors had signed a contract upon acceptance of their paper that they would make the data available to peers for reanalysis [12]. Wicherts et al. [13] subsequently showed that this sharing of data is related to the quality of the reporting of statistical results and the strength of evidence. In their study, they compared a subset of the articles used in Wicherts et al. [1], and found that articles from which data were shared contained relatively fewer reporting errors. Reporting errors are discrepancies between the reported *p* value and the recalculated *p* value based on the reported test statistic and degrees of freedom (*df*). Earlier, we [14] documented that more than half of the articles in psychology that involved the use of null hypothesis significance testing contained at least one such reporting error (see also [15], [16]). Wicherts et al. [13] found that articles from which no data were shared contained more reporting errors, more large reporting errors (differences in *p* larger than .01), and more reporting errors that changed the statistical conclusion, than articles from which the data were shared for reanalysis. Furthermore, *p* values are traditionally interpreted as the strength of evidence against the null hypothesis of no effect [17], and Wicherts et al. [13] found that the reluctance to share data was related to weaker evidence, as the mean *p* value within articles was higher on average (closer to .05) for articles from which the data were not shared, compared to the mean *p* value in articles from which the data were shared. This suggests that published statistical results are particularly hard to verify when re-analyses are more likely to lead to contrasting conclusions.

Reluctance to share data for independent reanalysis can therefore be seen as a Questionable Research Practice (QRP). In the current replicability crisis in psychology [18], [19] several practices related to the collection and analysis of data are identified as questionable, because the use of these practices might lead to an inflated Type I error rate and biased effect size estimates [20], [21]. John et al. [22] investigated the prevalence of different QRPs by surveying more than 2000 psychological researchers on whether they had ever used particular QRPs in their work. These QRPs ranged from failing to report all of a study's dependent variables (admitted by 63%) to falsifying data (admitted by .6%). Another QRP was “deciding whether to exclude data after looking at the impact of doing so on the results” (p. 525), which was admitted by 38% of the respondents. The exclusion of data in this ad hoc manner is questionable because both keeping and removing outliers can have a profound effect on the outcome of the analysis [23], [24]. Simmons et al. [21] noted that the handling of outliers in reaction time data in articles in the journal *Psychological Science* was quite inconsistent, suggesting that outlier exclusion is often subjective. What was considered too slow or too fast varied enormously, which enables self-serving justifications. On the other hand, even the common removal of outliers based on, say, absolute *Z* scores larger than a certain threshold value (common values of this threshold are 2 and 3) will inflate the Type I error rate [23] and is therefore not recommended.

The exclusion of data is also one of the few QRPs that can be detected by carefully reading a published article, as the removal of outliers and other data should be mentioned in the text in accordance with common guidelines. Notably, according to the APA publication manual, “omitting troublesome observations from reports to present a more convincing story is […] prohibited” (p. 12 [12]). Without preregistration of the analytic plan or the use of statistical protocols (which is uncommon in psychology), readers cannot distinguish ad hoc exclusion of outliers from exclusion on a priori grounds. Given that many psychologists admit to excluding data to see how it impacts the results, we consider the exclusion of outliers as an indicator of the *potential* use of *p-hacking* [25] or *significance chasing* [26] in null hypothesis significance testing.

Here, we investigate the relationship between outlier removal, reporting errors, and the strength of evidence against the null hypothesis in psychological articles. We compare two sets of articles from the same journals. The first set of articles reported the removal of outliers from the analyses, while the second set of articles reported no exclusion of outliers or other values. Like in our earlier study on data sharing [13], we analyzed data at the level of articles. We had three preregistered hypotheses: (1) Insofar that researchers remove outliers to get a significant *p* value (*p*<.05), we expected the average significant *p* value to be higher (closer to .05) in articles in which outliers were removed than in articles that reported no removal of outliers [13], [25]. (2) The removal of outliers is error prone because it involves multiple analyses, the results of which are easily confused in the process of analysis and reporting of results [14]. Therefore, we expected the number of reporting errors to be higher in articles that involved exclusion of outliers than in articles (in the same journal) that did not involve the exclusion of outliers. Furthermore, the proportion of articles with reporting errors concerned with *p*<.05 were expected to be higher for articles in which outliers are removed. Yet another reason to expect a relation between gross errors (i.e., misreporting of outcomes as being significant which appear not to be significant) is that this represents a QRP that has an estimated prevalence of 22% [22]. Given that the QRPs in John et al.'s [22] study formed a Guttman scale, gross errors can be seen as a good indicator of the use of other QRPs (including exclusion of outliers). (3) When the study is of high quality (i.e., is well designed and sufficiently powered), the original study outcome (without QRPs such as the ad hoc removal of outliers) will more likely be significant. Therefore, outlier removal to get a significant result is less needed for high quality studies than for studies of lower quality. Since power is positively related to sample size, we predicted the average sample size to be lower for articles in which outliers were removed compared to articles that reported no outlier removal. Another reason for expecting this relationship is that outliers exert relatively more influence on statistical results in small samples.

As we had clear hypotheses, followed the same procedure as Wicherts et al. [13] and agree with the current call for more confirmatory research [27], we preregistered our hypotheses and methods on the OSF Framework. Our preregistration document is available at https://openscienceframework.org/project/cBCfD/. We follow the registered procedure in our data collection and analyses. If we made unforeseen decisions or changes, or checked some alternative explanation with explorative analyses, we indicate that in the results section below.

## Method

### Selection of articles

In our study [23] about the removal of outliers and the inflation of the Type I error rate of independent samples *t* tests we systematically reviewed the current practice of outlier handling in psychology. To that end, we collected the 353 articles that contained the word ‘outlier’ for all articles published between 2001 and 2010 in the following journals: *Journal of Experimental Social Psychology* (JESP), *Cognitive Development* (CD), *Cognitive Psychology* (CP), *Journal of Applied Developmental Psychology* (JADP), *Journal of Experimental Cognitive Psychology* (JECP), and *Journal of Personality and Social Psychology* (JPSP). These journals represent major fields of inquiry within psychology and typically have high rejection rates. For each journal, we randomly selected 25 articles that contained the word “outlier” for closer examination. CD contained only 12 articles that used the term “outlier” in the given timeframe, and all 12 articles were examined. Of the full set of 137 articles, 108 reported to have removed outliers *before* conducting the analysis. In our preregistration we mention 106 articles of which outliers were removed before the actual analyses. However, checking the articles revealed one missing article and one duplicate article. The missing article was added and the duplicate was replaced with a new randomly drawn article, which resulted in a total sample size of 108 articles in which outliers were removed before the actual analyses. These 108 articles will be inspected further in the current study to see whether there outlier exclusion is related to the strength of the evidence against the null hypothesis, sample sizes, and the number of reporting errors.

To collect a comparable sample of articles in which outliers were not removed, we randomly selected 25 articles from each journal (12 from CD) in the same timeframe (2001 till 2010). The methodologies and types of analyses used in these different papers are quite comparable. We included only articles that did not report removing (exclusion; see also [28]) or adapting (transforming) any values, leading to a control sample of 88 articles. Table 1 gives the number of articles per journal.

**Table 1:** Number or articles per journal: (1) that mentioned ‘outlier’,(2) that were checked by Bakker & Wicherts [23], (3)that involved the removal of outlier(s) and the use of a t or F test, (4) in which no values were removed, and (5) in which no outliers were removed and a t or F test was used.

Journal*	Outlier mentioning	Outlier Removal	Removal and *F* or *t* test	No removal	No removal and *F* or *t* test
JESP	127	23	21	14	13
CD	12	9	6	8	6
CP	32	17	15	12	10
JADP	33	17	12	23	10
JECP	63	21	18	16	11
JPSP	86	21	20	15	11
Total	353	108	92	88	61

*JESP  =  Journal of Experimental Social Psychology, CD  = Cognitive Development, CP  =  Cognitive Psychology, JADP  =  Journal of Applied Developmental Psychology, JECP  =  Journal of Experimental Cognitive Psychology, and JPSP  =  Journal of Personality and Social Psychology.

### Collection of reported results

We collected all the completely (test statistic, *df*s, and *p* value) reported *t* and *F* tests (we did not collect the results from *χ^2^* tests as these tests are often less influenced by outliers) from each article with the *statcheck* package for R [29]. Statcheck automatically retrieves from the article's text all *t* and *F* test results that are reported in accordance with the APA style (e.g., “*F*(1,23)  = 4.45, *p* = .046”; [12]). After the collection of the results by statcheck, we searched all articles by hand to identify and include missed reported results that were not reported in APA style (e.g., because they reported an effect size between the test statistic and the *p* value). Only articles with at least one completely reported *t* or *F* test, with a reported *p* value smaller than .05 were included in our final sample. We collected by hand the total sample size of each separate study in each included article.

### Calculation of variables

Each reported *p* value (*p*<.05) was recalculated based on the reported test statistic and *df* with the statcheck package. For each article we calculated the median of the recalculated *p* values. The statcheck package also checks whether the reported *p* value is consistent with the recalculated *p* value, taking rounding into account [14]. All the errors found with the statcheck package were double-checked by hand, as for example one-sided tests might show an error in the automated procedure. We counted the total number of errors, the total number of large errors (i.e., those related to the 2^nd^ decimal), and the total number of gross errors (i.e., instances in which recalculation gave a non-significant result), and the total number of reported results per article. For each article we also calculated the median of the reported sample sizes.

### Analyses and Power

Our analytic plan followed our earlier paper on the relation between data sharing, strength of evidence, and quality of reporting of results [13]. Like in the earlier work, we analyzed the data at the level of articles. We did this because psychological articles often report numerous results that are dependent in rather intractable ways. Also, fine-grained analyses of single results are often impeded by a common lack of clarity about the precise analyses from which outliers were excluded and subjectivity in judging which analyses concern the main hypothesis. For comparing the magnitude of p-values and sample sizes across the two types of articles, we used a non-parametric Wilcoxon test and a bootstrap procedure. For this bootstrap procedure we randomly assigned each paper (and all the *p* values in it) to two groups (removal and no removal) on the basis of the number of articles in each condition, and compared the median *p* values in the two groups with a Wilcoxon test. This was repeated 100,000 times and the *W* values were collected to get an empirical null distribution, with which the *W* value of the actual difference could be compared. Moreover, we expected the number of errors to be higher in articles that reported the exclusion of outliers than in articles that did not report any removal of outliers (or other values). Because the number of errors in an article is dependent of the number of reported statistics, we used a negative binomial regression in which we controlled for the number of reported statistics (log) to predict the number of errors (for all, large, and gross errors separately).

In this study, we expected a comparable effect size as found in our earlier study concerning data sharing [13]. The number of included articles in our current study is approximately twice as large as the number of studies included in the study of Wicherts et al. and should therefore provide enough power. Furthermore, a pilot study [30] that compared psychological articles with and without reported data exclusion showed an effect size of approximately *d* = 0.5, which is comparable to common effect sizes found in psychology. An a priori simulation study showed that the power of the non-parametric Wilcoxon test was .9 when *α* = .05, *d* = 0.5 and 90 articles in both conditions.

## Results

### Final sample

**Figure 1:**
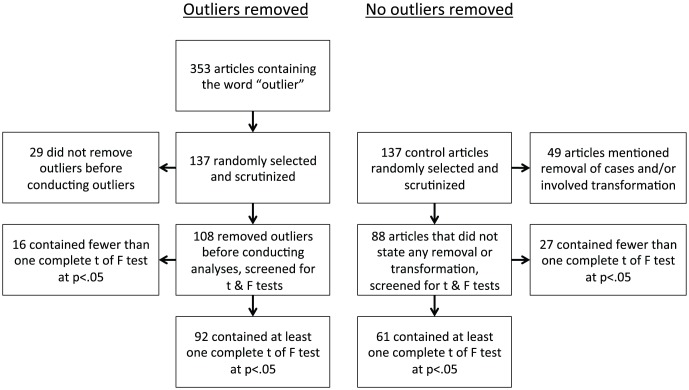
Flowchart of papers in the set of papers that stated outlier removal (left) and the set of papers that did not report any removal of outliers (right).

We followed our registered method to arrive at our final sample. The flow chart is given in Figure 1. Of the 108 articles in which outliers were removed, 92 articles reported at least one complete *t* or *F* test with *p*<.05. Of the 88 articles that reported no exclusion of values from the analysis, 61 reported at least one complete *t* or *F* test with *p*<.05. We collected 1781 statistical results reported as *p*<.05 from the articles in which outliers were removed. We collected 886 statistical results (*p*<.05) from the articles that reported no removal of outliers (or other values). Of all statistical results, 1847 (69%) were found by using the statcheck package and 820 (31%) while manually checking the results. The median number of reported statistics was 14 (*M* = 19.4) for articles in which outliers were removed and 12 (*M* = 14.5) for articles that reported no outlier removal. The number of reported significant results did not differ significantly between the two types of articles (non-registered; Wilcoxon rank sum test: *W* = 3202, *p* = .140). None of the journals showed a significant difference between the two types of articles in terms of the number of reported significant results per article (see Table 2). All data are available upon request.

**Table 2:** Median (mean) number of statistics per article for each journal and results of the Wilcoxon test.

Journal	Outlier removal	No outlier removal	Wilcoxon test
JESP	12 (14.00)	9 (11.69)	*W* = 163, *p* = .346
CD	10 (13.83)	6 (7.67)	*W* = 27.5 *p* = .127
CP	30 (34.13)	16.5 (16.60)	*W* = 99, *p* = .183
JADP	6 (12.08)	5 (5.50)	*W* = 71.5, *p* = .445
JECP	15 (14.17)	21 (18.45)	*W* = 70.5, *p* = .200
JPSP	23.5 (24.60)	22 (24.00)	*W* = 117, *p* = .772
Total	14 (19.36)	12 (14.52)	*W* = 3202, *p* = .140

### Strength of evidence (against the null hypothesis)

We expected higher median *p* values in articles in which outliers were removed compared to the median *p* values in articles that reported no exclusion of outliers. Unexpectedly, with the Wilcoxon test, we did not find a significant difference between the median *p* value in the articles in which outliers were removed (*Med*  = .0020, *M* = .0057) and the articles that reported no exclusion of outliers (*Med*  = .0029, *M* = .0063; *W* = 2785, *p* = .938). Similarly, the bootstrap procedure as described in the method section gave a *p* value of .469. Additional planned analyses also showed no significant differences between removal and non-removal of outliers at the journal level (see Table 3).

**Table 3:** Median (and mean) of the median p value per article for each journal and results of the Wilcoxon test.

Journal	Outlier removal	No outlier removal	Wilcoxon
JESP	.0054 (.0080)	.0094 (.0100)	*W* = 116, *p* = .484
CD	.0002 (.0020)	.0032 (.0058)	*W* = 8, *p* = .132
CP	.0017 (.0064)	.0022 (.0045)	*W* = 77, *p* = .935
JADP	.0005 (.0073)	.0046 (.0097)	*W* = 71, *p* = .497
JECP	.0001 (.0033)	< .0001 (.0024)	*W* = 124, *p* = .276
JPSP	.0024 (.0051)	.0046 (.0046)	*W* = 116, *p* = .823
Total	.0020 (.0057)	.0029 (.0063)	*W* = 2785, *p* = .938

**Figure 2:**
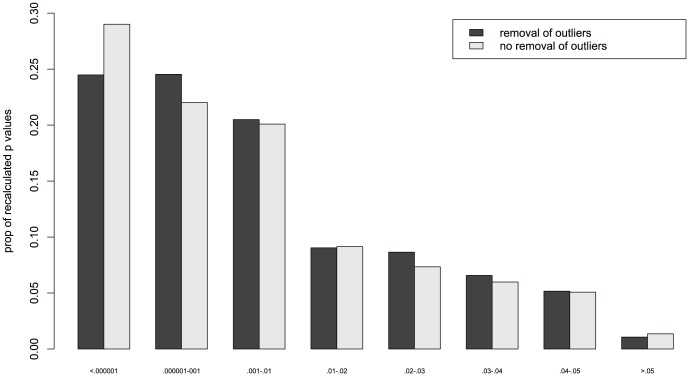
Distribution of *p* values reported as being significant (at *p*<.05) in 92 papers from which outliers were removed (N = 1781; in black) and in 61 papers that did not report any removal of outliers (N = 886; in grey).

Furthermore, we plotted the distribution of all the *p* values in Figure 2 for the articles in which outliers were removed and articles in which no outliers were removed separately [13]. Only for the smallest recalculated *p* values (<.000001) we witnessed a difference between the two distributions (Fisher-exact-test: *p* = .013; non registered comparison). A direct comparison (non registered) of all the *p* values which did not take into account dependencies within articles also showed no significant difference between the two types of results (those associated with removed outliers: *Med*  = .0012, *M* = .0097; no reported outlier removal: *Med*  = .0008, *M* = .0093): *W* = 820964.5, *p* = .088.

### Errors in the reporting of statistical results

In Table 4, we present the number of errors, large errors, and gross errors in each journal. Table 5 includes the number of articles with at least one error, at least one large error, and at least one gross error in each journal. We found approximately the same proportion of articles with at least one error (45%) or at least one gross error (15%) as we found earlier [13], [14]. Unexpectedly, negative binomial regressions that accounted for the number of test statistics showed that removal of outliers was not significantly predictive of all reporting errors, large reporting errors, and gross errors (see Table 6). Likewise, negative binomial regressions with the square root of the average *p* value per paper (a non-registered analysis), failed to show that removal of outliers was predictive for any kind of error. Fisher exact tests on the paper level (registered for when the proportion of papers with errors would be too small to perform negative binomial regressions) failed to corroborate the relation between removal of outliers and whether or not articles with at least one reporting error, large reporting error, or gross reporting error (*p* = .870, *p* = .339, *p*>.99, respectively).

**Table 4:** Number of statistics, number of errors, number of large errors, and number of gross errors for each journal separately for articles in which outliers were removed and for articles that did not report any removal of outliers.

Outliers	N Statistics	N Error	N Large Error	N Gross Error
JESP	294	10 (3.4%)	3 (1.0%)	0 (0.0%)
CD	83	13 (15.7%)	1 (1.2%)	1 (1.2%)
CP	512	27 (5.3%)	4 (0.8%)	6 (1.2%)
JADP	145	22 (15.2%)	3 (2.1%)	5 (3.4%)
JECP	255	6 (2.4%)	2 (0.8%)	1 (0.4%)
JPSP	492	33 (6.7%)	3 (0.6%)	6 (1.2%)
Total	1781	111 (6.2%)	16 (0.9%)	19 (1.1%)
No outliers				
JESP	152	10 (6.6%)	2 (1.3%)	2 (1.3%)
CD	46	0 (0.0%)	0 (0.0%)	0 (0.0%)
CP	166	24 (14.5%)	12 (7.2%)	4 (2.4%)
JADP	55	4 (7.3%)	0 (0.0%)	2 (3.6%)
JECP	203	19 (9.4%)	2 (1.0%)	2 (1.0%)
JPSP	264	10 (3.8%)	0 (0.0%)	1 (0.4%)
Total	886	67 (7.6%)	16 (1.8%)	11 (1.2%)

**Table 5:** Number of articles, number articles with at least one error, number of articles with at least one large error, and number of articles with at least one gross error for each journal separately for articles in which outliers are removed and for articles that did not report any removal of outliers.

Outliers	N Journal	N Error	N Large Error	N Gross Error
JESP	21	7 (33%)	3 (14%)	0 (0%)
CD	6	4 (67%)	1 (17%)	1 (17%)
CP	15	11 (73%)	3 (20%)	5 (33%)
JADP	12	6 (50%)	2 (17%)	3 (25%)
JECP	18	6 (33%)	2 (11%)	1 (6%)
JPSP	20	8 (40%)	3 (15%)	4 (20%)
Total	92	42 (46%)	14 (15%)	14 (15%)
No outliers				
JESP	13	3 (23%)	1 (8%)	2 (15%)
CD	6	0 (0%)	0 (0%)	0 (0%)
CP	10	6 (60%)	3 (30%)	3 (30%)
JADP	10	2 (20%)	0 (0%)	1 (10%)
JECP	11	8 (73%)	2 (18%)	2 (18%)
JPSP	11	8 (73%)	0 (0%)	1 (9%)
Total	61	27 (44%)	6 (10%)	9 (15%)

**Table 6:** Results of the negative binomial regressions for all errors, large reporting errors, and gross errors.

Predictor	Parameter (SE)	*p*
All reporting errors (range 0–10)		
Intercept	−2.309 (0.464)	<.001
Outliers removed (1) or not (0)	−0.019 (0.264)	.943
Log (No. of test statistics)	0.892 (0.156)	<.001
Neg. Binomial parameter	0.764 (0.185)	
Large reporting errors (range 0–8)		
Intercept	−3.734 (0.948)	<.001
Outliers removed (1) or not (0)	−0.565 (0.515)	.273
Log (No. of test statistics)	0.888 (0.320)	.006
Neg. Binomial parameter	0.272 (0.128)	
Gross errors (range 0–2)		
Intercept	−4.386 (0.862)	<.001
Outliers removed (1) or not (0)	−0.178 (0.418)	.670
Log (No. of test statistics)	1.004 (0.273)	<.001
Neg. Binomial parameter	1.72 (2.28)	

*Note*: Negative binomial regressions (N = 153) of the number of reporting errors per paper on the log of the number of test statistics and whether or not outliers were removed. Analyses were estimated with the glm.nb function from the MASS package in R (SPSS 20.0 gave similar results). Natural log transformations were used to improve predictors' normality.

### Sample Size

We expected the average sample size to be lower for articles that reported the exclusion of outliers compared to articles that reported no removal of outliers. We compared these conditions as had been planned with the Wilcoxon test and the bootstrap procedure as described in the methods section. The sample sizes in two articles were insufficiently described and so we had to remove these articles from the following analyses. Unexpectedly, articles in which outliers were removed did not involve significantly smaller sample sizes (*Med*  = 82, *M* = 119.01) than articles that reported no removal of outliers (*Med*  = 86.5; *M* = 154.57; *W* = 2601.5, *p* = .625). The bootstrap procedure gave comparable results (p = .311). Table 7 gives the results per journal. However, ignoring dependencies and analyzing all sample sizes of all the studies together (a non-registered analysis) did show a significant difference in sample size between studies from articles in which outliers are removed (*Med*  = 66; *M* = 101.36) and from articles that reported no outlier removal (*Med*  = 81.5; *M* = 126.40; *W* = 11505, *p* = .008). Also a bootstrap procedure showed a significant difference (*p* = .003; non-registered analysis) for this comparison.

**Table 7:** Median (mean) of the median sample size per article for each journal and results of the Wilcoxon test.

Journal	Outlier removal	No outlier removal	Wilcoxon
JESP	90 (116.57)	72 (79.88)	*W* = 170.5, *p* = .228
CD	101 (99.00)	101.25 (126.08)	*W* = 14, *p* = .589
CP	41 (54.89)	46.5 (42.28)	*W* = 58.5, *p* = .777
JADP	115 (184.25)	134.25 (221.85)	*W* = 62, *p* = .923
JECP	88 (157.78)	73 (179.91)	*W* = 103, *p* = .857
JPSP	81 (98.40)	109 (263.73)	*W* = 66, *p* = .069
Total	82 (119.01)	86.5 (154.57)	*W* = 2601.5, *p* = .625

### Alternative explanations: incomplete disclosure

Our planned analyses failed to corroborate the expected differences in median *p* value, reporting errors, and sample size. A reason might be that the removal of outliers was not clearly reported in the articles in our control group, notwithstanding that APA guidelines (APA, 2010) stipulate that reporting of exclusions should be reported. Recent results suggest that not all exclusions of data are reported in psychological articles. LeBel et al. [31] surveyed authors of papers in various top psychology journals about the reporting of methods and exclusion of values, and found that 11% of the researchers admitted that they did not fully disclose all excluded values in their paper. If this is also true in our current sample of articles, the group of articles that did not report any exclusions of outliers might be contaminated with studies in which these values actually were removed from the analyses. Such contamination might influence our results. To check whether the group of papers without reported exclusions may have included some unreported exclusions, we checked all 34 articles of this group that contained at least one *t* test. Specifically, we checked whether the sample size described in these articles matched the reported *df* of the relevant *t* tests. When values (cases) are removed from the analysis this might lead to inconsistencies between the reported sample size and the reported *df*, although such inconsistencies could also arise because of erroneous reporting of the *df* or because of unreported missing data (we verified that missing data was not mentioned in the articles when retrieving the sample size descriptions). We choose to focus here on *t* tests, as the relation between the *df* of the *t* test and the sample size is quite clear. MB and JMW independently rated the 34 articles and agreed on 125 (81%) of the 154 checked *t* tests. The differences between the raters were solved by discussion. In total, we found the *df*s of 35 of the 154 *t* tests (23%) to be inconsistent with the reported sample size (after checking for potential dropout or missingness). In total, we found 14 out of 34 articles (41%) to contain at least one discrepancy between the reported sample size and the reported *df*. Although these discrepancies between sample size and *df* may be due to other factors (e.g., unreported missing data, or misreporting of the *df*), these results do suggest that exclusions of data (because of outliers and for other reasons) are often not reported in psychological articles. This is in line with results by LeBel et al. [31] and supports our alternative explanation that we failed to find the hypothesized differences because the set of control papers was contaminated by results that also involved the exclusion of data. Note that we did not find significant differences in median *p* value, number of errors, or sample size, between the articles in which we found or did not found a discrepancy, but the sample size and therefore the power for these comparisons were very low. Furthermore, exclusion of the 14 articles that showed a discrepancy from our final data set did not alter the original results.

## Discussion

We hypothesized that outlier exclusion would be associated with relatively high *p* values (below the .05 threshold), more reporting errors, and smaller sample sizes and studied this in a sample of psychology papers. We found a proportion of reporting errors comparable with our earlier results [13], [14]. Nevertheless, none of our preregistered hypotheses were confirmed. We failed to find a significant difference between the articles in which outliers were removed and articles that reported no outlier removal in the median *p* value, number of errors, or the median sample size. An unregistered comparison of the sample sizes (while ignoring dependencies within articles) showed that sample sizes were indeed lower in articles in which outliers were removed, compared to articles that reported no outlier removal. Furthermore, we found a difference between articles in which outliers were or were not removed in the proportion of very small *p* values (<.000001). Since our preregistered analyses failed to confirm our hypotheses, these results may suggest that removing outliers is unrelated to reporting errors and strength of evidence (against the null hypothesis).

However, there might be other explanations. First, the effect could be smaller than expected, which might have resulted in a power that was too low to detect an effect in the current sample. Second, taking all the reported statistical results in an article-level analysis is not a very precise measurement as articles often contain many results that differ in importance (e.g., the results of a manipulation check versus the results of the analysis to test the main hypothesis). Therefore, the data may have contained too much noise to measure the difference between the two types of articles. For a more fine-grained analysis, future research could use the *p* curve method [25] which focuses only on the results of the main analysis. We note however that it is often difficult when reading psychological articles to distinguish between the core analyses and more exploratory analyses among the typically dozen or so presented results. Then again, Wicherts et al. [13] used the same method as we used in the current study to compare articles of which the data were or were not shared and they found clear differences between the two types of papers.

Another explanation might be that articles in which nothing is reported about removing outliers, actually did involve the removal of outliers (or other data points). Recently, LeBel et al. [31] asked 347 authors to disclose design specifications and almost half of the authors replied and disclosed publicly the requested information. Of those who responded to LeBel et al. (supposedly a biased sample leading to underestimates), 11.2% admitted that they had not fully disclosed all excluded values in their article. Thus our control group of articles in which the exclusion of outliers was not mentioned could also have contained articles in which outliers were indeed removed. To check whether this lack of reported exclusions could have influenced our results, we checked the consistency between the sample size and the reported *df* of *t* tests in articles that did not report any data removal or missingness. In 41% of the articles we checked, we found at least one discrepancy between sample size description and the *df*s. This suggests that our sample of control articles was contaminated with articles in which data were excluded without mention. Given that unreported exclusion of data may not always be visible by comparing the *df*s and reported sample sizes, it is quite possible that unreported exclusion of data is even more common in psychological research than our current results suggest. Not much is known about the prevalence of inconsistencies between sample size and *df*s in the literature, but they may well be similar to those found by related methods [14]–[16], [32], [33]. Rossi [33] found 4 cases of inconsistent *df*s in his sample of 46 *t* and *F* tests. Future research should address the prevalence of misreporting of *df*s and/or the reasons why so often the described *df*s are inconsistent with the reported sample size. In addition, future research of the relation between exclusion and the magnitude of the *p* values should involve the use of a less contaminated control sample. This could be achieved by checking with the authors whether they have excluded any values, or by only using the papers that used the “21-words solution” [34] in which authors explicitly state “We report how we determined our sample size, all data exclusions (if any), all manipulations, and all measures in the study.” (p. 4). Another solution might be to use only articles that fully disclosed that they had not excluded any values on PsychDisclosure.org [31], or to use future articles from Psychological Science, which installed a disclosure policy related to the exclusion of data in early 2014 [35].

Although we did not find evidence for the relationship between outlier removal and reporting errors and strength of evidence (against the null hypothesis), other factors might have concealed this relationship. Nevertheless, outliers should be carefully handled, as the unjust removal of extreme values can increase the Type I error rate. Therefore, other methods that are less influenced by possible outliers, like non-parametric or robust statistics [36], should be considered Furthermore, to prevent the (unconscious) subjective removal of outliers, an outlier handling protocol could be written down before seeing the data. The preferred practice is to explicate the handling of outliers in advance as part of the study registration [23]. Above all, researchers should be transparent in their articles about the exclusion of data.
